# Clinical profiles, incidence and predictors of early neonatal mortality at Mbarara Regional Referral Hospital, south-western Uganda

**DOI:** 10.1186/s12887-024-05014-4

**Published:** 2024-08-23

**Authors:** Lydia Kyasimire, Leevan Tibaijuka, Moses Ochora, Musa Kayondo, Elias Kumbakumba, Josephine Nantongo, Stella Kyoyagala

**Affiliations:** 1 Department of Paediatrics and Child Health, Mbarara University of Science and Technology, P.O. Box 1410, Mbarara, Uganda; 2 Department of Obstetrics and Gynaecology, Mbarara University of Science and Technology, Mbarara, Uganda; 3 Department of Paediatrics and Child Health, Mbarara Regional Referral Hospital (MRRH), Mbarara, Uganda

**Keywords:** Early Neonatal Mortality, Clinical profiles, Predictors of early neonatal mortality

## Abstract

**Background:**

The current neonatal mortality rate in Uganda is high at 22 deaths per 1000 live births, while it had been stagnant at 27 deaths per 1000 live births in the past decade. This is still more than double the World Health Organization target of < 12 deaths per 1,000 live births. Three-quarters of new born deaths occur within the first week of life, which is a very vulnerable period and the causes reflect the quality of obstetric and neonatal care. At Mbarara Regional Referral Hospital (MRRH), the modifiable contributors and predictors of mortality remain undocumented, yet neonates make the bulk of admissions and contribute significantly to the overall infant mortality rate. We therefore examined the clinical profiles, incidence and predictors of early neonatal mortality of neonates admitted at MRRH in south-western Uganda.

**Methods:**

We conducted a prospective cohort study at the Neonatal Unit of MRRH between August – November, 2022 among neonates. We consecutively included all live neonates aged < 7 days admitted to neonatal unit and excluded those whose outcomes could not be ascertained at day 7 of life. We obtained baseline data including; maternal social-demographic and obstetric information, and performed neonatal physical examinations for clinical profiles. We followed up neonates at 24 and 72 h of life, and at 7 days of life for mortality. We summarized the clinical profiles and incidence of mortality as frequencies and percentages and performed modified Poisson regression analysis to identify the predictors of early neonatal mortality.

**Results:**

We enrolled 384 neonates. The majority of neonates were in-born (68.5%, *n* = 263) and were admitted within 24 h after birth (54.7%, *n* = 210). The most common clinical profiles at admission were prematurity (46%, *n* = 178), low birth weight (LBW) (44%, *n* = 170), sepsis (36%, *n* = 139), hypothermia (35%, *n* = 133), and birth asphyxia (32%, *n* = 124). The incidence of early neonatal mortality was at 12.0%, 46 out of the 384 neonates died. The predictors of early neonatal mortality were hypothermia, [adjusted Risk Ratio: 4.10; 95% C.I (1.15–14.56)], birth asphyxia, [adjusted Risk Ratio: 3.6; 95% C.I (1.23–10.73)] and delayed initiation of breastfeeding, [adjusted Risk Ratio: 7.20; 95% C.I (1.01–51.30)].

**Conclusion:**

Prematurity, LBW, sepsis, birth asphyxia and hypothermia are the commonest admission diagnoses. The incidence of early neonatal mortality was high, 12.0%. We recommend targeted interventions by the clinical care team at MRRH to enable timely identification of neonates with or at risk of hypothermia to reduce incidence of adverse outcomes. Intrapartum care should be improved in order to mitigate the risk of birth asphyxia. Breastfeeding within the first hour of birth should be strengthened were possible, as this is associated with vast benefits for the baby and may reduce the incidence of complications like hypothermia.

## Introduction

The neonatal period, which lasts from the day of birth through the first 28-days of life, is when a child’s survival and health are most at risk [1]. Neonatal mortality accounts for 57% of the global infant mortality and 40% of all under-5 mortality [2].

The first week of life is a very vulnerable period which accounts for nearly three-quarters of new-born deaths, with one-third occurring on the first day of life [2, 3]*.* Sub-Saharan Africa has the greatest mortality rates and a tenfold risk of neonatal death compared to the developed countries [2]. Similar to the Uganda’s stagnant neonatal mortality rate over the past 10 years, sub-Saharan Africa has a neonatal mortality rate of 27 deaths per 1,000 live births since 2019 [3]. The neonatal mortality rate in Uganda has however improved to 22 deaths per 1000 live births [4]. This is however, more than twice the Sustainable Development Goal target for neonatal mortality set at less than 12 deaths per 1,000 live births [5].

The maternal risk factors like extremes of age—teenagers and advanced maternal age (> 35 years), multiparity and short birth intervals, as well as delayed initiation of breastfeeding are among the factors reported to have increased the risk of neonatal mortality [6]. The other risk factors include place of birth, neonatal characteristics like gestational age, birth weight and Apgar score [7, 8].

Scant setting specific data on neonatal characteristics and predictors of mortality in the LMICs impacts the efforts directed towards improving neonatal outcomes [9, 10], which would be crucial in directing the timely essential interventions to reduce early neonatal mortality [11]. This study therefore aimed to describe the clinical profile of neonates and determine the incidence and predictors of early neonatal mortality among neonates admitted to the MRRH neonatal unit.

## Materials and methods

### Study setting and design

We conducted a prospective cohort study of live neonates admitted to the neonatal Unit of Mbarara Regional Referral Hospital (MRRH) from August, 2022 to November, 2022.

MRRH is a government funded public hospital, situated in Mbarara city in southwestern Uganda, 260 kms from Kampala, the capital city of Uganda. The hospital has a catchment population of 3.4 million people, and a 494-bed capacity. The hospital serves as a teaching hospital for Mbarara University of science and Technology (MUST) and other tertiary health training institutions in the region. MRRH receives patients from all the districts of Ankole and Kigezi subregions in southwestern Uganda, and part of greater Masaka subregions in central Uganda including Mbarara, Isingiro, Bushenyi, Buhweju, Ibanda, Kiruhura, Mitoma, Ntungamo, Rwamapara, Sheema, Rubirizi, and Lyantonde. The hospital provides services to patients from 2 refugee camps (Nakivaale, Oruchinga) who are from Rwanda, Burundi, Congo, Somalia, Sudan, and Ethiopia.

The obstetrics and gynecology department at MRRH houses the maternity ward and a high risk obstetrics unit that is located about 20 m from the neonatal unit, while the operating theatre is approximately 40–50 m from the neonatal unit. The high-risk obstetrics unit manages mothers with complicated pregnancies. The maternity ward conducts 800–1000 deliveries every month [7]. The Obstetrics and Gynaecology department comprises 14 obstetricians, 33 residents, 12 intern doctors, and 19 midwives (with 10 working on any given day).

Sick neonates are transferred to the neonatal unit by the caretaker or a midwife from theatre or labour suit. There are no facilities for providing oxygen support or warmth while the neonate is in transit to the neonatal unit.

The Paediatric ward is managed by a team of 10 paediatricians, 20 paediatric residents, 8 intern doctors and 10 nurses. The neonatal unit is staffed with 2 paediatricians, and 6 nurses. The nurses work in 8-to-12-h shifts, and on average each shift has 2 nurses. The unit has 3 intern doctors and 3 paediatric resident doctors on rotational basis. The neonatal unit functions as a level II unit, however receives neonates that require advanced respiratory and cardiovascular support. According to hospital records, the neonatal unit admits close to 3,000 neonates every year (60% of overall paediatric admissions), therefore approximately 250 sick neonates are admitted every month.

About two-thirds of the neonates admitted to this new born unit are born at MRRH, and the remaining third are either referred in from other medical centres or are brought in directly from the local populations. The neonatal unit is a 49-bed capacity unit, divided into 4 sections, the high dependency unit, 26 cots (for both very sick term and preterm babies), 8-bed capacity Kangaroo mother care room for stable preterm and a unit for stable terms with 15 beds. The unit sometimes admits up to thrice its capacity, with neonates sharing infant warmers and cots. The neonatal unit had recently received a donation of medical equipment, and currently has 14 neonatal phototherapy machines, 4 radiant warmers, 8 infusion pumps, 5 monitors. Along with 14 back-up oxygen concentrators, there is a supply of medical oxygen in oxygen cylinders. The unit does not have a mechanical ventilator and Continuous Positive Airway Pressure machines (CPAP), as well as blood gas machines. The unit uses bubble CPAP with cold unblended oxygen, which is locally developed for neonates with respiratory distress syndrome (RDS), as the main stay of treatment for RDS. Exogenous surfactant is not available, due to resource limitations. The nursing services offered include provision of intravenous antibiotics (commonly ampicillin and gentamycin), intravenous fluids, phototherapy and nasal gastric tube feeding. Mothers feed their babies on a 2-hourly basis. Neonates once discharged are followed up routinely through the neonatal clinic, every Thursday.

### Study population and eligibility criteria

This prospective cohort study included neonates admitted to the neonatal unit of MRRH. We included all live neonates within the first 7 days of life, including neonates with congenital anomalies. Both in-born and out-born neonates were included in this study. Runaways and self-discharges that could not be contacted on the 7th day of life were considered as lost to follow up and were excluded from the final analysis.

### Sample size estimation and sampling

The sample size was calculated using Open Epi version 3, specifically employing the sample size determination for cohort studies. The calculations were based on a previous study conducted among neonates admitted at Mulago National Referral Hospital in Uganda, with the proportion of mortality among out-born neonates (34%) and proportion of mortality among in-born neonates (20%) [8], we estimated a total sample size of 384 (128 out born and 256 inborn) neonates. We assumed 80% power, 95% confidence interval and the ratio of the unexposed (inborn neonates) to exposed (out born neonates) of 2. We therefore consecutively enrolled 384 live neonates.

### Study variables

#### Clinical profiles

The clinical profiles were the neonatal clinical characteristics and diagnoses obtained at the time of admission. These included hypothermia (axillary temperature < 36.5°c, taken with a lithium battery-operated digital thermometer), hypoglycaemia (random blood sugar < 2.2 mmol/dl) in a capillary blood sample from a heel prick for blood glucose testing using an electronic glucometer [12], admission weight, gestation age, neonatal sepsis, as well as jaundice, abnormal bleeding, congenital anomaly, and birth asphyxia (5 min APGAR score less than 7)/ Hypoxic ischemic encephalopathy (HIE), prematurity, respiratory distress syndrome. The gestation age was calculated from the last normal menstrual period, or estimation from obstetrics ultra sound scan, if available and a Ballard score was used to assess neonates with either a last normal menstrual period or obstetric scan. A neonate was considered preterm if delivered before 37 completed weeks of gestation, and a birth weight below 2.5 kg was considered low birth weight. These were defined as per Integrated Management of childhood illnesses (IMCI) [12, 13].

Neonatal diagnoses were defined as 1) RDS—presence of any of: fast breathing, grunting, subcostal and intercostal recession, cyanosis and reduced air entry in bilateral lung fields starting in the first four hours of life, chest radiographs (x-rays) were not done [12, 13].; 2) birth asphyxia—a 5-min Apgar score of less than 7 [14]; 3) jaundice/hyperbilirubinemia—yellowing of eyes and/or body requiring phototherapy or serum bilirubin levels above 15 mg/dL according to the WHO’s Integrated Management of Childhood Illnesses (IMCI) algorithm [12]; 4) sepsis—we diagnosed sepsis clinically, by examining for one or more of the clinical characteristics listed; change in level of activity, bulging fontanelle,history of convulsions, feeding difficulty, temperature ≥ 37.5 °C or < 35.5 °C, fast breathing / respiratory rate ≥ 60 bpm, severe chest in drawing, grunting and/or.

cyanosis according to the WHO’s Integrated Management of Childhood Illnesses (IMCI) algorithm [12], blood cultures were not done. The cause of death was defined as the clinical condition directly and immediately leading to the death of the neonate as documented in the neonate’s medical chart/records.

#### Predictor variables

Neonatal predictors; gestational age, sex, Apgar score at 5 min, birth weight, breastfeeding status, temperature. Maternal and obstetric predictors included maternal age, level of education, marital status, occupation, number of antenatal care (ANC) contacts, mode of delivery, parity, and maternal HIV serostatus.

#### Outcome/ dependent variable

The outcome variable of interest was early neonatal death/ mortality. We defined the early neonatal death as death of a neonate any time from admission to 7 days after birth. We obtained information on early neonatal death from the neonate’s chart for the neonates who were still admitted in the neonatal unit, and followed up by phone for those discharged home.

### Recruitment and follow-up of participants

We approached mothers/caretakers at the point of admission, after the neonate had been initiated on care and stabilised, to obtain permission to participate in the study. Alongside the clinical care team, we performed a complete evaluation of the neonate, including history and the physical exam. We approached mothers/caretakers only for information missing from the patient charts. We followed up the enrolled neonates at 24 and 72 h, and on the 7th day of life to ascertain outcome of interest, whether the neonate was dead or alive, through observation and chart review. We followed up the neonates discharged home via a phone call. We collected and managed data using a pretested data collection tool in Research Electronic Data Capture (Redcap) [15]. The study team research assistants used android phones with pre-installed Redcap to collect data. We checked collected data for completeness every day. The research assistants were experienced nurses in regard to new born care, and had attained a certificate of good clinical practice.

### Data management and analysis

Data were exported to STATA 17 (StataCorp, College Station, Texas, USA) for cleaning and analysis. We reported the baseline socio-demographic, obstetric, and neonatal characteristics of the participants using proportions. We described the clinical profiles as frequencies and percentages, and presented in a graph. We reported the incidence of early neonatal deaths as a percentage of the total number of neonates enrolled to the end of the follow up period of the study. To determine the predictors of early neonatal mortality, we used the modified Poisson regression analysis to perform both univariable and multivariable analysis and reported crude and adjusted risk ratios respectively. In the final multivariable model, we included all exposure variables with a *p*-value < 0.2 at univariable analysis, as well as age, based on biological plausibility. To assess collinearity in our multivariable model, we employed the variance inflation factor (VIF) method, considering VIF > 5 as indicative of collinearity. The predictors of early neonatal mortality in the multivariable model were considered statistically significant if variables had a *p*-value < 0.05.

## Results

**Fig. 1:**
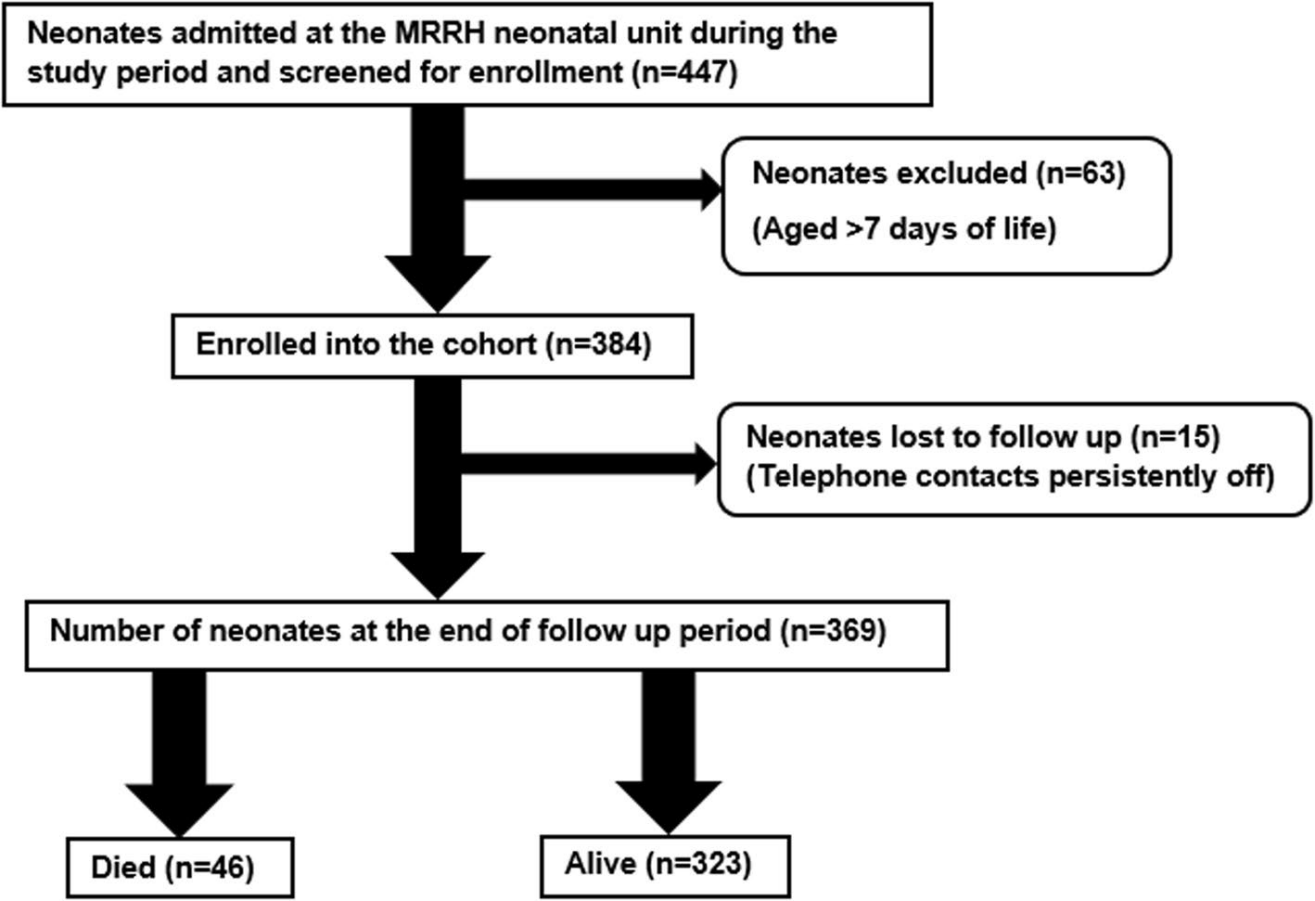
Study flow chart showing recruitment, exclusion, and follow-up of participants, at Mbarara Regional Referral Hospital, August – November, 2022

We screened 447 neonates for inclusion into the study between August – November, 2022, and enrolled 384 neonates after excluding neonates beyond the first week of life. We excluded 63 neonates who were aged beyond the first 7 days of life. We lost 15 neonates (3.9%) to follow up, and analysed 369 neonates for incidence and predictors of mortality (Fig. 1).

### Maternal socio-demographic and obstetric characteristics

The mean age of the mothers was 26 years, with majority (73.4%) aged between 20 to 34 years. Less than 10% of the mothers had tertiary level education and 3.4% had not attained any level of education. Majority (95%) of the mothers were married and more than half (57.8%) had no employment. Majority of the mothers had a parity of 2 to 4 (44%), a third of them were first time mothers. Majority (70%) of the mothers had 4 or more antenatal care contacts, but majority (66%) had their first antenatal care contact in the second trimester of pregnancy. Additionally, 10% of the mothers were HIV positive, and majority carried a singleton pregnancy. Just over half of the mothers (58%) had normal vaginal deliveries. The common pregnancy complications were spontaneous preterm labour (22%), preeclampsia (23%), and antepartum hemorrhage (17%) (Table 1).

**Table 1:** Socio-demographic and obstetric characteristics of mothers whose neonates were admitted at MRRH between August – November, 2022 (*n* = 384)

Variable	Frequency (%)
Maternal age in years	
< 20	53 (13.8%)
20–34	282 (73.4%)
> 35	49(12.8%)
Formal Education level	
Uneducated	13 (3.4%)
Primary	166 (43.3%)
Secondary	155 (40.5%)
Tertiary	49 (12.8%)
Married	365 (95.1%)
House hold monthly income (Uganda shillings)	
250,000–500,000	66 (17.2%)
50,000–250,000	147 (38.3%)
< 50,000	171 (44.5%)
Parity	
1	139 (36.2%)
2–4	169 (44.0%)
≥ 5	76 (19.8%)
Number of antenatal care visits	
None	4 (1.1%)
1–3	114 (28.6%)
≥ 4	270 (70.3%)
Gestation at first antenatal care visit (*n* = 380)	
1st trimester	120 (31.6%)
2nd trimester	251 (66.1%)
3rd trimester	5(1.3%)
Twin pregnancy	48 (12.6%)
HIV sero-positive	32 (8.4%)
Obstetric complications	
Spontaneous preterm labour	88 (22.9%)
Preeclampsia	23 (6.0%)
Antepartum haemorrhage (placenta previa and abruption)	17 (4.4%)
No obstetric complication	256(66.7%)
Mode of delivery	
Caesarean section	156 (40.6%)
Vertex vaginal delivery	223 (58.1%)
Breech vaginal delivery	5 (1.3%)
Referred from another health unit	103(26.8%)

### Baseline characteristics of the neonates

Of the 384 neonates enrolled during the study period, just over half (53.6%) of the babies were term (> 37 weeks), and had a normal birth weight (50.5%). The most prevalent gender was male, with 56.7%. Only 11.6% of the neonates had Apgar < 7 at 5 min. It was noted that 62% of the neonates had not breast fed within the initial hour of life. Majority of the neonates (68.5%) were delivered at MRRH, while the rest were born in other health facilities (Table 2).

**Table 2:** Baseline characteristics of neonates admitted at MRRH between August – November, 2022 (*n* = 384)

Variable	Frequency (%)
Gestational age (weeks)	
Preterm (< 37)	178 (46.4%)
Term (≥ 37)	206 (53.6%)
Neonatal age at admission	
Within 24 h	210 (54.7%)
24–72 h	133 (34.7%)
> 72 h < 7 days	41 (10.7%)
Sex of the neonate	
Female	166(43.3%)
Male	217(56.7%)
Birth weight (kilograms)	
Big baby (≥ 4)	20 (5.2%)
Normal (2.5 to < 4)	194(50.5%)
Low birth weight ( < 2.5)	170(44.3%)
Apgar score at 5 min < 7	41 (11.6%)
Breast feeding initiated in the first 1 h	62(37.6%)
Place of birth	
In-born (born at MRRH maternity ward)	263 (68.5%)
Out-born (born at other health facilities and referred in)	121(31.5%)

### Clinical profiles of the neonates admitted at MRRH

**Fig. 2:**
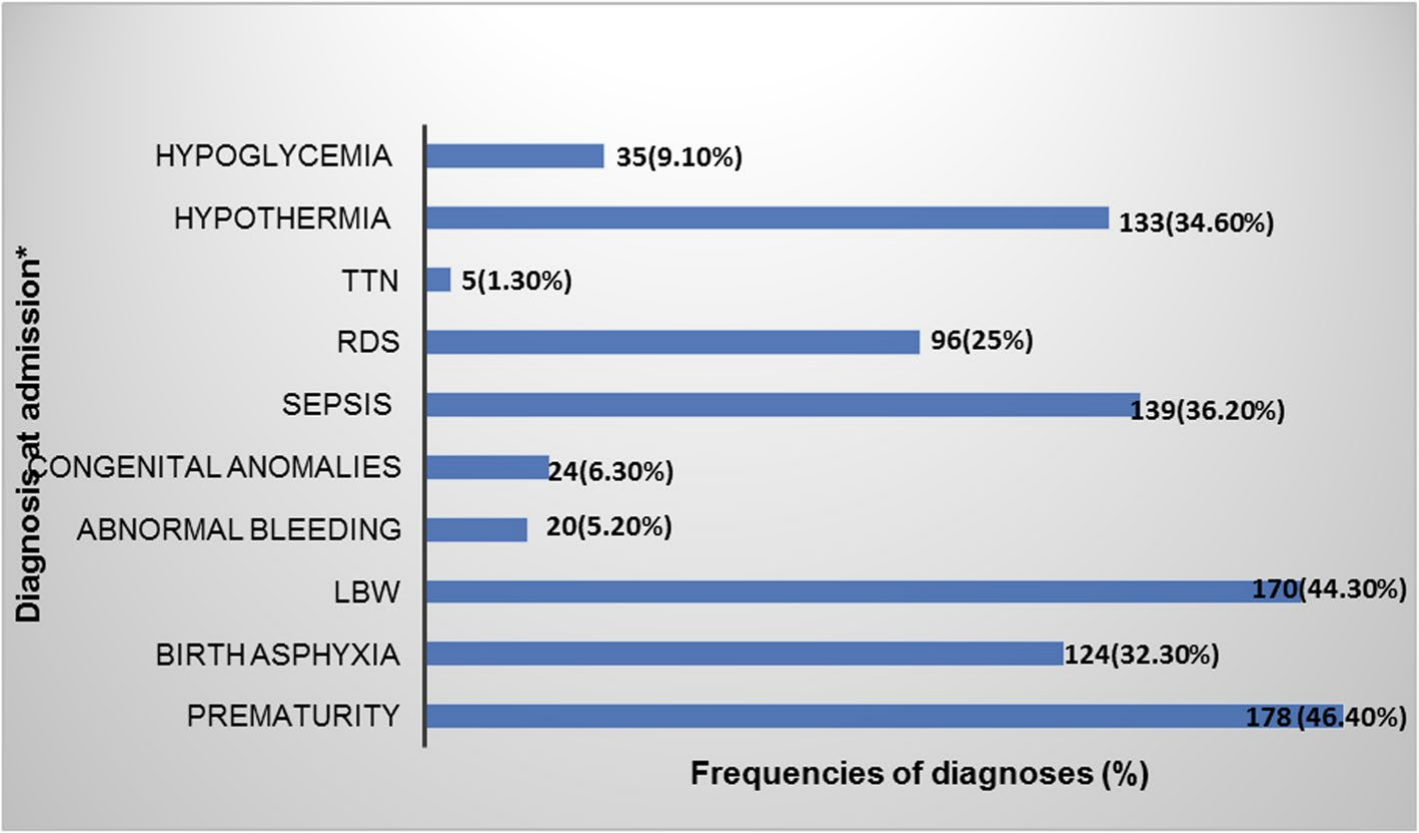
Clinical profiles of the neonates admitted to MRRH neonatal unit from August- November, 2022 (*n* = 384); *the clinical profiles were not mutually exclusive; RDS: Respiratory distress syndrome; TTN: transient tachypnea of the newborn; LBW: Low birth weight

Close to half of the babies enrolled, (46%) were preterm neonates and 44% were low birth weight. The other diagnostic categories at admission included sepsis (36%), hypothermia (35%), birth asphyxia (32%), and respiratory distress syndrome (RDS) 25%. Additional admission clinical conditions included jaundice (10%), hypoglycemia (9%) and congenital anomalies (6%). Of the 24 neonates admitted with congenital anomalies, 4 had spina bifida, clubfoot (*n* = 4), omphalocele (*n* = 4), gastroschisis (*n* = 2), congenital heart disease—Tetralogy of Falot (*n* = 1), congenital hydrocephalus (*n* = 5), anencephaly (*n* = 1), cleft lip and palate (*n* = 3 (Fig. 2).

### Characteristics of in-born versus out-born neonates admitted to MRRH

There was a significant difference in characteristics of in-born versus out-born neonates in terms of preterm rupture of membranes, pre-eclampsia/eclampsia, caesarean delivery, birth asphyxia and neonatal jaundice. More in-born neonates were delivered from mothers with pre-eclampsia/eclampsia (8.4% versus 0.8%), were born by caesarean Sect. (52.1% versus 15.7%) and suffered birth asphyxia (36.1% versus 24.0%). However, preterm rupture of membranes (5.7% versus 12.4%) and neonatal jaundice (8.0% versus 15.7%) were more common among out-born neonates (Table 3).

**Table 3:** Characteristics of in-born versus out-born neonates admitted to MRRH between August – November, 2022 (*n* = 384)

Variable	In-born (*N* = 263)	Out-born (*N* = 121)	*P*-value
	**n/N (%)**	**n/N (%)**	
Multiple pregnancy	28 (10.7%)	20 (16.5%)	0.110
Preterm rupture of membranes	15 (5.7%)	15 (12.4%)	0.023*
Spontaneous preterm labour	58 (22.1%)	30 (24.8%)	0.550
Preeclampsia/eclampsia	22 (8.4%)	1 (0.8%)	0.004*
Antepartum hemorrhage	14 (5.3%)	3 (2.5%)	0.210
Malpresentation	14 (5.3%)	3 (2.5%)	0.210
Chorioamnionitis	2 (0.8%)	2 (1.7%)	0.420
Mode of delivery			< 0.001*
Caesarean section	137 (52.1%)	19 (15.7%)	
Vaginal delivery	126 (47.9%)	102 (84.3%)	
Preterm births (< 37 weeks)	138 (52.5%)	68 (56.2%)	0.500
Sex of the neonate			0.094
Female	106 (40.5%)	60 (49.6%)	
Male	156 (59.5%)	61 (50.4%)	
Birth weight (kilograms)			0.220
Big baby (≥ 4)	12 (4.6%)	8 (6.6%)	
Normal (2.5- < 4)	127 (48.3%)	67 (55.4%)	
Low birth weight (< 2.5)	124 (47.1%)	46 (38.0%)	
Apgar score at 5 min < 7	31 (12.2%)	10 (10.2%)	0.600
Respiratory distress syndrome	69 (26.2%)	27 (22.3%)	0.410
Birth Asphyxia	95 (36.1%)	29 (24.0%)	0.018*
Hypothermia	95 (36.1%)	38 (31.4%)	0.370
Hypoglycemia	28 (10.6%)	7 (5.8%)	0.120
Neonatal jaundice	21 (8.0%)	19 (15.7%)	0.021*
Neonatal sepsis	97 (36.9%)	42 (34.7%)	0.680

^*^p < 0.05

### Early neonatal mortality at MRRH

**Fig. 3:**
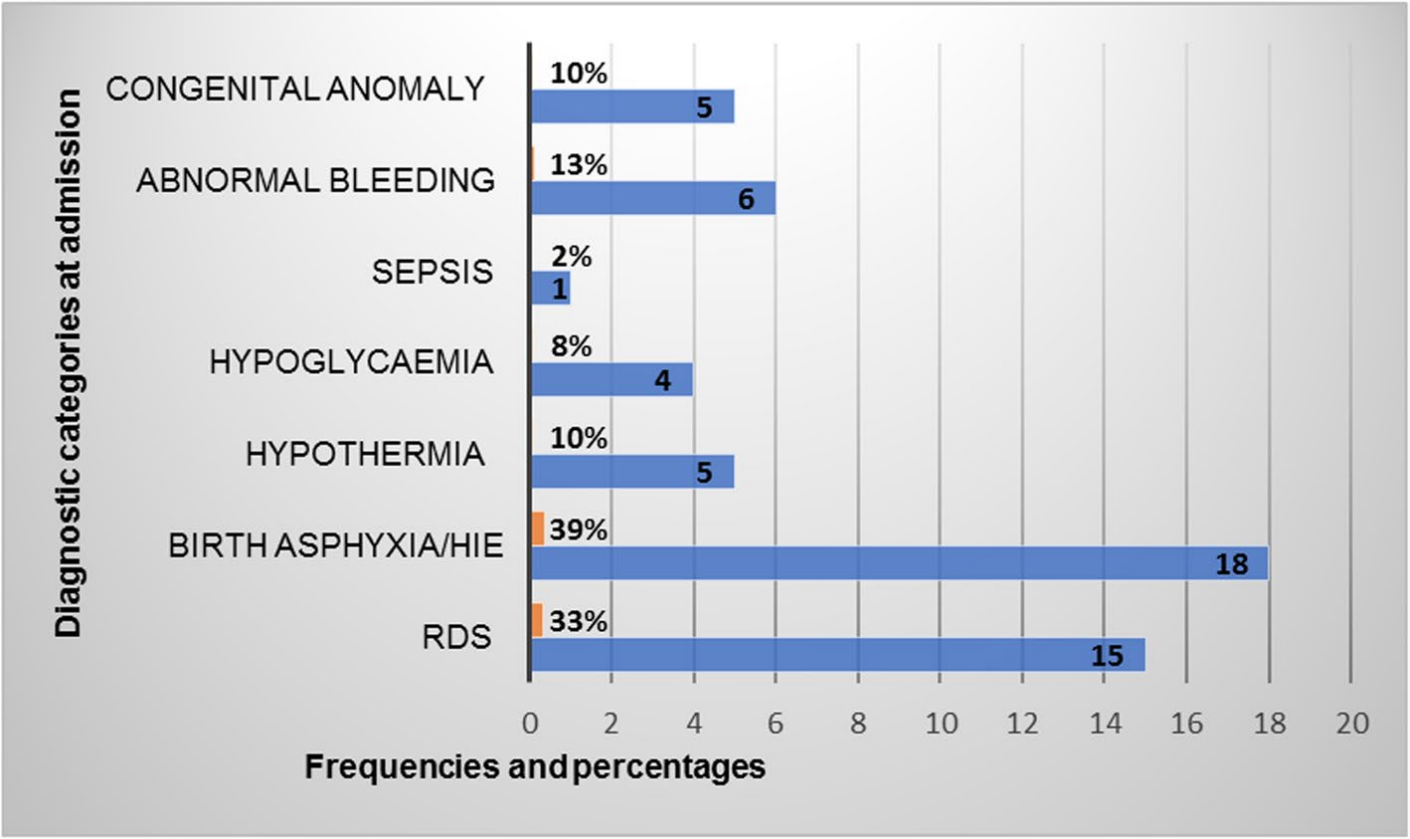
Causes associated with early neonatal mortality at MRRH (*n* = 46). HIE: Hypoxic Ischemic encephalopathy; RDS: Respiratory distress syndrome

Overall, 46/384 (12.0%) neonates died in the first week of life. The out born neonates had a mortality of 11.6% (14/121), while the inborn neonates had a mortality of 12.2% (32/263). Of the neonates who died, 12 (26%) died in the first 24 h of life, 15 (33%) died between 24 to 72 h, while the rest died after the first 72 h. Over half of the neonates who died (56.5%, *n* = 26) were preterm neonates (gestational age < 37 weeks). Of these, 8 were late preterm (34- < 37 weeks), 3 (moderate preterm, 32- < 34 weeks), 9 (very preterm, 28- < 32 weeks) and 6 (early preterm, < 28 weeks). Majority of the neonates died following complications of birth asphyxia (39%, *n* = 18) and respiratory distress syndrome (RDS) (32%, *n* = 15). Sepsis contributed to the least number of neonatal deaths (*n* = 1). Five of the neonates with congenital anomalies died, they included; congenital heart disease—Tetraloty of Falot (*n* = 1), gastroschisis (*n* = 2), anencephaly (*n* = 1), and congenital hydrocephalus (*n* = 1) (Fig. 3).

### Predictors of early neonatal mortality among neonates admitted at the neonatal unit of MRRH between August – November, 2022

At multivariable analysis; hypothermia, birth asphyxia and late breast-feeding initiation (1 h after delivery) were independent predictors of early neonatal mortality. Hypothermia increased the risk of early neonatal mortality by four times, [adjusted Risk Ratio: 4.10; 95% C.I (1.15–14.56), *p* = 0.029], birth asphyxia increased the risk of early neonatal mortality by 3.6 times, [adjusted Risk Ratio: 3.6; 95% C.I (1.23–10.73), *p* = 0.020] and delayed initiation of breastfeeding was associated with seven-fold increase in risk for early neonatal mortality, [adjusted Risk Ratio: 7.20; 95% C.I (1.01–51.30), *p* = 0.049] (Table 4).

**Table 4:** Multivariable analysis of predictors of early neonatal mortality among neonates admitted at MRRH

Variable	Died (*N* = 46)	Univariable analysis		Multivariable analysis	
	**n/N (%)**	**Crude RR (95% C.I)**	*P*-value	**Adjusted RR (95% C.I)**	*P*-value
**Maternal age (years)**					
< 20	6(13.0%)	0.9 (0.42–2.12)	0.882	0.93 [0.25,3.46]	0.911
20–34	34 (73.9%)	Ref		Ref	
> 34	6 (13.0%)	1.08 (0.48–2.44)	0.841	-	-
**Formal Education**					
≥ Secondary	26 (56.5%)	Ref		Ref	
≤ Primary	20 (43.5%)	0.66(0.37–1.18)	0.161	0.82[0.37,1.83]	0.630
**Antenatal care contacts**					
≥ 4	27 (58.7%)	Ref		Ref	
< 4	19 (41.3%)	1.68 (0.98–2.90)	0.059	1.15[0.48,2.75]	0.749
**Mode of delivery**					
Vaginal births	29 (63.0%)	Ref		Ref	
C-section	17 (37.0%)	1.08(0.62,1.90)	0.770	1.02[0.39,2.70]	0.964
**Place of birth**					
Inborn	32 (69.6%)	Ref		Ref	
Out born	14 (30.4%)	0.91 (0.50–1.65)	0.767	1.13[0.35,3.61]	0.835
**Gestational age (weeks)**					
Term (≥ 37)	26 (56.5%)	Ref		Ref	
Preterm (< 37)	20 (43.5%)	1.49 (0.86–2.58)	0.150	0.60[0.09,4.01]	0.595
**Sex of neonate**					
Female	18 (40.0%)	Ref		Ref	
Male	27 (60.0%)	1.12 (0.64–1.97)	0.677	1.50[0.35,6.39]	0.580
**Respiratory distress syndrome**					
No	24 (52.2%)	Ref		Ref	
Yes	22 (47.8%)	2.65 (1.56–4.50)	< 0.001**	1.13[0.50,2.57]	0.772
**Hypothermia**					
** No**	**15 (32.6%)**	**Ref**		**Ref**	
** Yes**	**31 (67.4%)**	**3.83 (2.15–6.81)**	< 0.001**	**4.10 [1.15,14.56]**	0.029*
**Hypoglycemia**					
No	38 (82.6%)	Ref		Ref	
Yes	8 (17.4%)	2.04 (1.03, 4.00)	0.046*	0.79[0.13,4.76]	0.796
**Breastfeeding initiation**					
** Early (< 1 h)**	**1 (6.3%)**	**Ref**		**Ref**	
** Late (> 1 h)**	**15 (93.8%)**	**9.29 (1.25–69)**	0.006*	**7.20[1.01,51.30]**	0.049*
**Birth weight (kilograms)**					
≥ 2.5		Ref		Ref	
< 2.5		1.14 (0.66–1.95)	0.644	0.96[0.07,12.54]	0.974
**Birth asphyxia**					
** No**	**15 (32.6%)**	**Ref**		**Ref**	
** Yes**	**31 (67.4%)**	**4.43(2.49,7.89)**	< 0.001**	**3.63[1.23,10.73]**	0.020*

*RR *Risk Ratio*, Ref *Reference group*, CI *Confidence Interval

^*^p < 0.05

^**^p < 0.001

## Discussion

This prospective cohort study examined the clinical profile of neonates and determined the incidence and predictors of early neonatal mortality among live neonates admitted to the neonatal unit at a tertiary hospital in a low-resource setting in southwestern Uganda. The results showed that the bulk of neonatal admissions had a diagnosis of prematurity, low birth weight, sepsis, hypothermia and birth asphyxia. Every 1 in 10 neonates died in the early neonatal period, and hypothermia, birth asphyxia and late initiation of breastfeeding were independent predictors of early neonatal mortality. Overall, these findings highlight the need for targeted implementation of the intrapartum and neonatal care protocols. This will ensure timely identification of the neonates at increased risk of adverse outcomes while in-utero and during labor, and hence prevent birth asphyxia. These findings highlight the need to strengthen the warm chain by optimizing early interventions that correct and prevent complications associated with preterm and low birth weight neonates, especially hypothermia. These interventions include thermal control of the delivery room temperature during deliveries, use of dry and pre-warmed towels, immediate skin to skin contact of baby and mother, as well resuscitation of neonates under warmers [16]. These practices are vital to reduce early neonatal mortality especially in the low resource settings.

In the current study, preterm and low birth weight neonates accounted for almost half of the neonatal admissions, followed by hypothermia, sepsis and birth asphyxia. Our findings are consistent with previous studies conducted in sub-Saharan Africa including at Kiwoko hospital in Uganda and in Ghana, with prematurity responsible for almost half of the admissions [17, 18]. The significant number of preterm and low birth weight neonates admitted highlight the need for clinical care preparedness and investment in feasible practices like administration of antenatal corticosteroids to high risk pregnancies (as practiced at our study site) and essential neonatal care interventions through providing the warm chain including kangaroo mother care, timely resuscitation of the neonates and encouraging early initiation of breast feeding [7].

Neonatal sepsis was noted in one third of the neonatal admissions. Our results are consistent with studies done in other low resource settings and for example in Kenya and other sub-Saharan African countries [3, 19]. This similarity could be attributable to the similar poor socio-economic status, with similar challenges in accessing equitable health care for the mothers and their neonates and harmful practices that predispose to sepsis. For example the level of neonatal care provided by these facilities is perhaps limited by absence of advanced diagnostic facilities, or practices that abate sepsis in the neonates. In low resource settings, the high prevalence of sepsis may be due to multiple risk factors attributable to the suboptimal obstetric care for the mothers and their new-borns, coupled with harmful traditional therapeutic practices to the neonates like superficial skin cuts, uvulectomy, false teeth extraction, use of herbal remedies for skin care and application of cow dung to the cord, among others [20]. Routine hand-washing before handling of the baby by caregivers prevents sepsis, but may not be optimally practiced in our setting [21, 22]. Mothers with risk factors predisposing their neonates to sepsis should be identified early, and preventative measures like administration of prophylactic antibiotics taken to reduce occurrence of neonatal sepsis.

We found that 12.0% of the neonates died. This finding is consistent with other studies in the low resource settings including Mulago (central Uganda), and Kenya, and India that reported the early neonatal mortality rate ranging from 10–12% [23–25]. This similarity of results could be because the study settings are similar in socio-economic status, with similar health care challenges. For example limited resources in terms of funds and personnel to adequately provide at minimum level 2 neonatal care—through changes in practice, routine audits, procurement of basic equipment, training and dedicating of neonatal unit staff as noted from other regional hospital in Uganda and other low income countries [26–28]. The main causes of early neonatal death are consequences of maternal obstetric complications like obstructed labour, cord prolapse, preterm rupture of membranes, antepartum haemorrhage and complicated deliveries, among others [3]. This is a reflection on the quality of obstetric care mothers received.

We report a similar incidence of mortality for both the in-born and out-born neonates; this is consistent with a study in India [23]. Studies at Kiwoko and Mulago hospitals, in Central Uganda, however reported higher mortality in out-born neonates—up to twice that of in-born neonates [8, 17]. This disparity could be explained by the advancement in care for mothers and neonates in the past 10 years with strengthening of support supervision to the lower level health facilities and improving the referral systems, mainly encouraging in-utero transfer of high risk pregnancies for delivery at centres with expertise and equipment to provide optimal care for the mothers and neonates. Additionally, this could be explained by the significant number of in-born neonates that were the result of high risk pregnancy conditions like preeclampsia/eclampsia which were associated with delivery of very sick neonates with life threatening conditions like birth asphyxia which were significant contributors to mortality in our study.

Our study found more than half of the mortalities to have occurred in the first 72 h which is in keeping with literature from other studies. Generally, studies report that more than 3 quarters of neonatal deaths occur within the first week of life [23, 29, 30]. Evidence from literature demonstrates that severe respiratory issues, hypothermia, hypoglycaemia, a delayed foetal to neonatal circulatory transition, and respiratory distress of prematurity are the commonest causes of early new born deaths [25, 30]. In this study, a third of the neonates had hypothermia, asphyxia or RDS. Furthermore, one third of the mothers in this study presented with obstetrics complications. Evidence from literature shows that maternal obstetric complications increase the risk of early neonatal deaths [31].

The independent predictors for early neonatal mortality were hypothermia, birth asphyxia and delayed initiation of breastfeeding. Similar to our study findings, prior studies have reported hypothermia on admission has been reported to be associated with early neonatal mortality [32–35]. Hypothermia is a major clinical concern in the preterm, low birth weight and/ or sick neonates due to a large surface area to body mass ratio, reduced subcutaneous fat, a thin and immature skin, a high body water content, as well as immature metabolism [25]. In our study, 73.9% (*n* = 99) with hypothermia were preterm neonates. Hypothermia affects multiple body systems including the cardiopulmonary (bradycardia, apnoea), the central nervous system (lethargy, poor feeding), the vascular system, as well the metabolic system, causing hypoglycaemia, hypoxia and metabolic acidosis, all which cause death [36]. Insufficient knowledge as opposed to lack of resources is the root cause of hypothermia, according to WHO [37]. Studies have demonstrated that hypothermia is a risk factor for early neonatal mortality [38], and more to this, the European guidelines on neonatal resuscitation emphasise good thermal control during resuscitation, so as to optimise good outcomes [16]. This implies that early recognition and prevention of hypothermia in these babies with focused management will reduce the cases of hypothermia, and subsequently the incidence of neonatal mortality at MRRH.

Birth Asphyxia accounted for one third of our admissions and predicted early neonatal mortality—subsequently contributing to a third of the mortality in our study. Our results are similar to studies done in Uganda [8, 17, 32], and Ethiopia [10]. Birth asphyxia is a major cause of neonatal mortality in resource constrained areas for various reasons that include inadequate obstetrics health coverage, social cultural norms that cause delays in accessing emergency obstetrics and neonatal care [39]. The 3 delays in accessing obstetric care [40], inconsistencies in management of labour, system factors like poor resource allocations and management, high levels of illiteracy and shortage in health workers are important indirect causes of birth asphyxia in resource limited settings [41]. These results highlight the need to strengthen intrapartum care through labor monitoring and timely detecting of fetal distress, prioritise and build capacity for new-born resuscitation and initial new-born care following birth. Promoting early referrals of mothers with obstetric complications, and early seeking of care by mothers, will help curb the rate of birth asphyxia.

Delayed initiation of breastfeeding (> 1 h) increases the risk of early neonatal mortality sevenfold in our study. This is in agreement with several studies done in Uganda [7, 42], India [43], and Ethiopia [10, 35], that report increased odds of death for neonates with delayed initiation of breastfeeding. Neonatal mortality can be reduced by 20 to 30% if early initiation of breastfeeding is practiced [43, 44]. Therefore, programmes and activities that promote early initiation of breastfeeding will go a long way in curbing early neonatal mortality.

The strength of this study is that it was conducted as a prospective study of both inborn and out-born neonates at a tertiary referral centre—with a large number of neonates admitted and referred in from surrounding health facilities with no outward transfers. We were therefore able to determine predictors of mortality from the identified variables, among the neonates. The other strength is that the attrition rate was minimised by the short follow up period. However, this study was limited by the fact that it was carried out in a single centre, therefore the results may not be representative of the rest of the regions in Uganda.

## Conclusions

The commonest cause of neonatal admissions were low birth weight, prematurity, sepsis, hypothermia and birth asphyxia. The early neonatal mortality rate was very high, 12.0%, with similar mortality among the inborn and out born neonates. The causes of death were mainly birth asphyxia and RDS. Hypothermia, birth asphyxia and delayed initiation of breastfeeding were the predictors of early neonatal mortality in our study.

Our study findings highlight the need of clinical care teams to identify neonates with or at risk of hypothermia, and optimise early interventions to correct or prevent it. This includes focused assessment of neonates, proper use of available facilities (infant warmers, clean and dry linen), and promotion of Kangaroo mother care, with continuous monitoring, until resolution. There is need for integrated maternal-paediatric care when planning deliveries of mothers with high-risk pregnancies in order to provide timely emergency neonatal care. Maternal and paediatric clinical care teams should ensure that neonates without severe morbidity should be supported to breastfeed within the first hour life. Continued support supervision to peripheral facilities should be strengthened in order to ensure timely referral of mothers with high-risk pregnancies, in-utero referral of likely preterm deliveries, and safe transfer of sick neonates.

## Data Availability

The datasets used during this study are available from the corresponding author upon reasonable request.

## Abbreviations

CPAP: Continuous positive airway pressure
IMCI: Integrated management of childhood illnesses
KMC: Kangaroo mother care
LBW: Low birth weight
LMIC: Low and middle income countries
LNMP: Last normal menstrual period
MMR: Maternal mortality ratio
MRRH: Mbarara Regional Referral Hospital
MUST: Mbarara University of Science and Technology
RDS: Respiratory distress syndrome
TTN: Transient tachypnoea of the newborn
WHO: World Health Organisation
